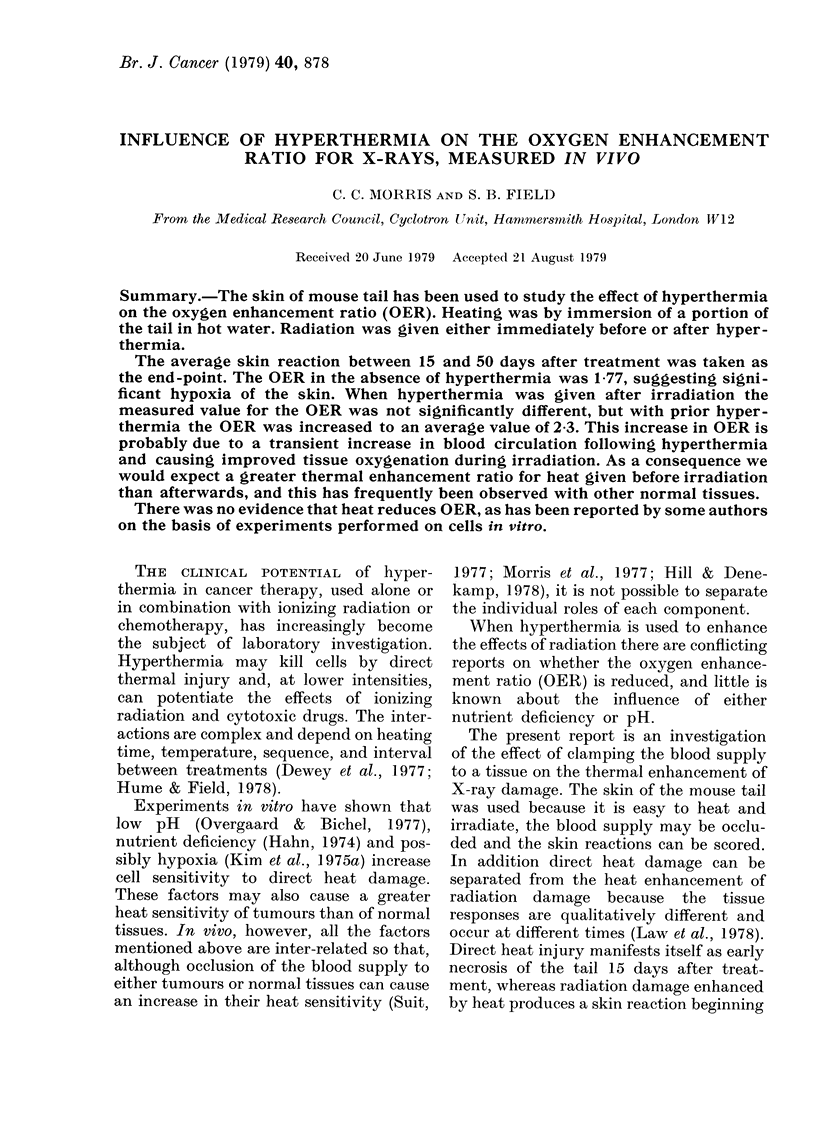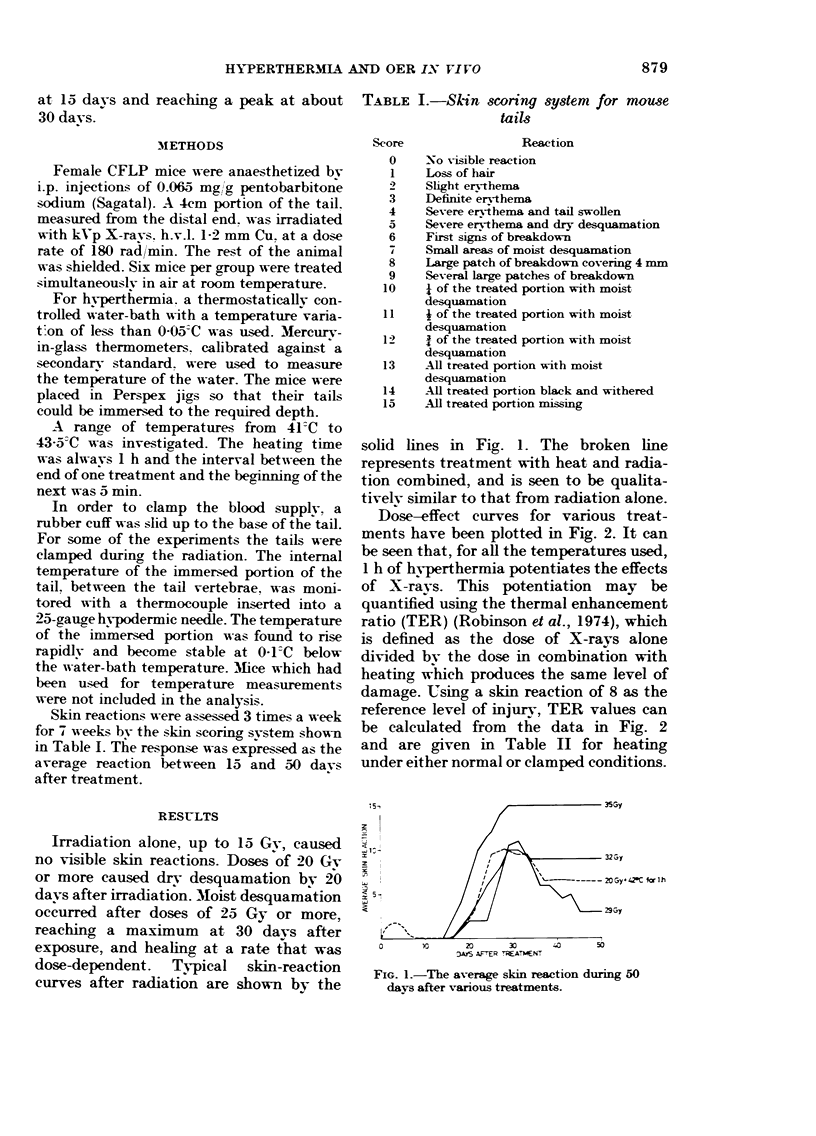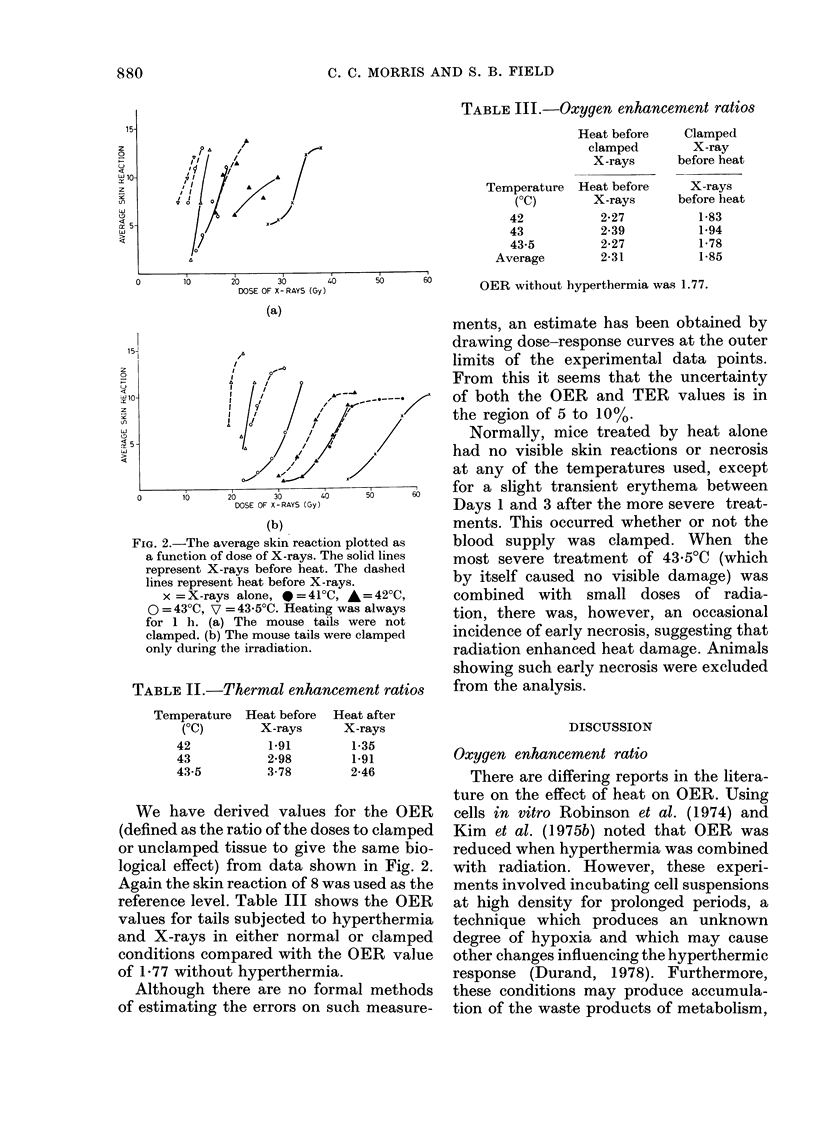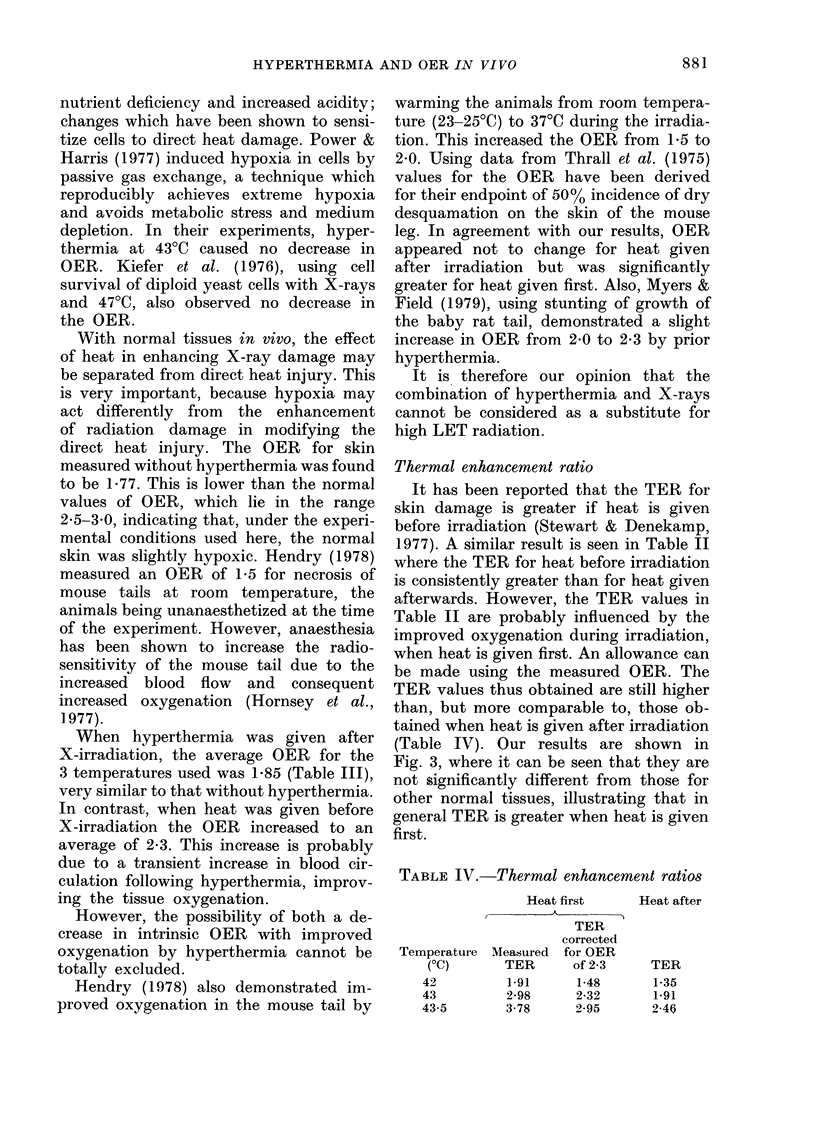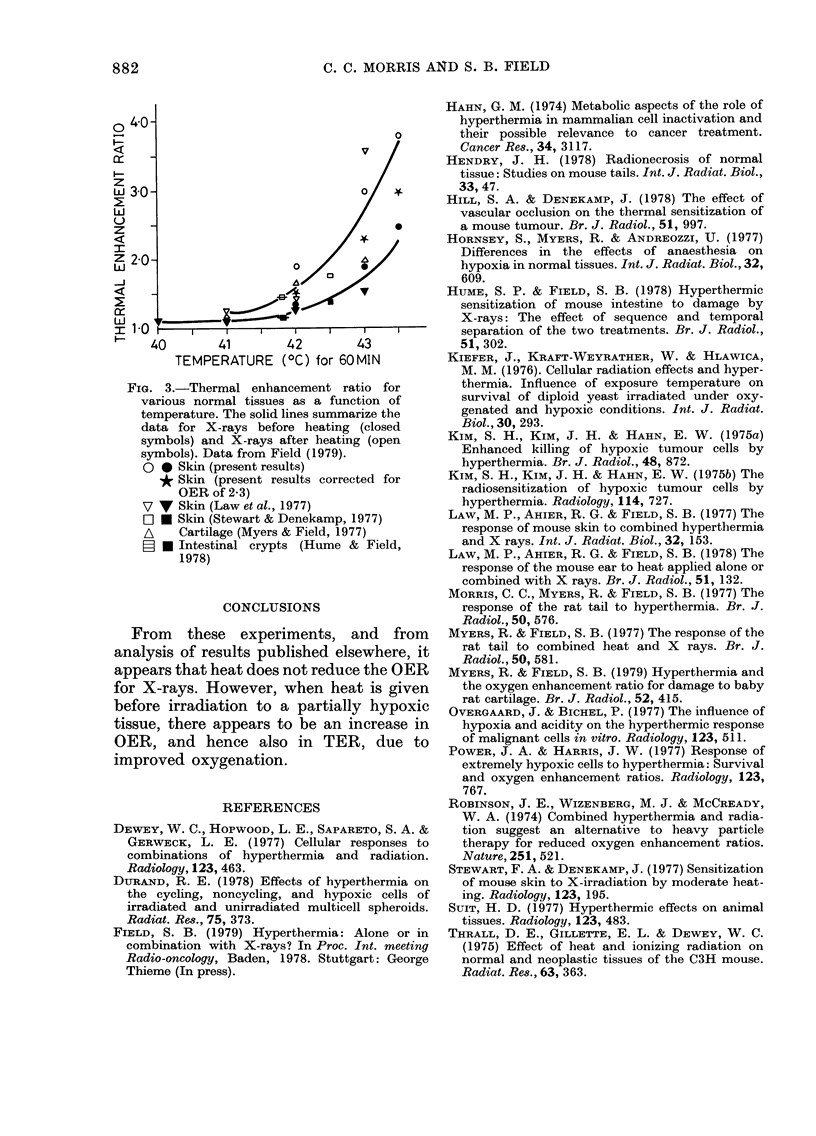# Influence of hyperthermia on the oxygen enhancement ratio for x-rays, measured in vivo.

**DOI:** 10.1038/bjc.1979.280

**Published:** 1979-12

**Authors:** C. C. Morris, S. B. Field

## Abstract

The skin of mouse tail has been used to study the effect of hyperthermia on the oxygen enhancement ratio (OER). Heating was by immersion of a portion of the tail in hot water. Radiation was given either immediately before or after hyperthermia. The average skin reaction between 15 and 50 days after treatment was taken as the end-point. The OER in the absence of hyperthermia was 1.77, suggesting significant hypoxia of the skin. When hyperthermia was given after irradiation the measured value for the OER was not significantly different, but with prior hyperthermia the OER was increased to an average value of 2.3. This increase in OER is probably due to a transient increase in blood circulation following hyperthermia and causing improved tissue oxygenation during irradiation. As a consequence we would expect a greater thermal enhancement ratio for heat given before irradiation than afterwards, and this has frequently been observed with other normal tissues. There was no evidence that heat reduces OER, as has been reported by some authors on the basis of experiments performed on cells in vitro.


*Br. J. Cancer* (1979) **40**, 878

### INFLUENCE OF HYPERTHERMIA ON THE OXYGEN ENHANCEMENT RATIO FOR X-RAYS, MEASURED *IN VIVO*

C. C. MORRIS AND S. B. FIELD

*From the Medical Research Council, Cyclotron Unit, Hammersmith Hospital, London W12*

Received 20 June 1979 Accepted 21 August 1979

**Summary.—The skin of mouse tail has been used to study the effect of hyperthermia on the oxygen enhancement ratio (OER). Heating was by immersion of a portion of the tail in hot water. Radiation was given either immediately before or after hyperthermia.**

**The average skin reaction between 15 and 50 days after treatment was taken as the end-point. The OER in the absence of hyperthermia was 1·77, suggesting significant hypoxia of the skin. When hyperthermia was given after irradiation the measured value for the OER was not significantly different, but with prior hyperthermia the OER was increased to an average value of 2·3. This increase in OER is probably due to a transient increase in blood circulation following hyperthermia and causing improved tissue oxygenation during irradiation. As a consequence we would expect a greater thermal enhancement ratio for heat given before irradiation than afterwards, and this has frequently been observed with other normal tissues.**

**There was no evidence that heat reduces OER, as has been reported by some authors on the basis of experiments performed on cells *in vitro*.**

THE CLINICAL POTENTIAL of hyperthermia in cancer therapy, used alone or in combination with ionizing radiation or chemotherapy, has increasingly become the subject of laboratory investigation. Hyperthermia may kill cells by direct thermal injury and, at lower intensities, can potentiate the effects of ionizing radiation and cytotoxic drugs. The interactions are complex and depend on heating time, temperature, sequence, and interval between treatments (Dewey *et al.*, 1977; Hume & Field, 1978).

Experiments *in vitro* have shown that low pH (Overgaard & Bichel, 1977), nutrient deficiency (Hahn, 1974) and possibly hypoxia (Kim *et al.*, 1975*a*) increase cell sensitivity to direct heat damage. These factors may also cause a greater heat sensitivity of tumours than of normal tissues. *In vivo*, however, all the factors mentioned above are inter-related so that, although occlusion of the blood supply to either tumours or normal tissues can cause an increase in their heat sensitivity (Suit, 1977; Morris *et al.*, 1977; Hill & Denekamp, 1978), it is not possible to separate the individual roles of each component.

When hyperthermia is used to enhance the effects of radiation there are conflicting reports on whether the oxygen enhancement ratio (OER) is reduced, and little is known about the influence of either nutrient deficiency or pH.

The present report is an investigation of the effect of clamping the blood supply to a tissue on the thermal enhancement of X-ray damage. The skin of the mouse tail was used because it is easy to heat and irradiate, the blood supply may be occluded and the skin reactions can be scored. In addition direct heat damage can be separated from the heat enhancement of radiation damage because the tissue responses are qualitatively different and occur at different times (Law *et al.*, 1978). Direct heat injury manifests itself as early necrosis of the tail 15 days after treatment, whereas radiation damage enhanced by heat produces a skin reaction beginning at 15 days and reaching a peak at about 30 days.

### METHODS

Female CFLP mice were anaesthetized by i.p. injections of 0.065 mg/g pentobarbitone sodium (Sagatal). A 4cm portion of the tail, measured from the distal end, was irradiated with kVp X-rays, h.v.l. 1·2 mm Cu, at a dose rate of 180 rad/min. The rest of the animal was shielded. Six mice per group were treated simultaneously in air at room temperature.

For hyperthermia, a thermostatically controlled water-bath with a temperature variation of less than 0·05°C was used. Mercury-in-glass thermometers, calibrated against a secondary standard, were used to measure the temperature of the water. The mice were placed in Perspex jigs so that their tails could be immersed to the required depth.

A range of temperatures from 41°C to 43·5°C was investigated. The heating time was always 1 h and the interval between the end of one treatment and the beginning of the next was 5 min.

In order to clamp the blood supply, a rubber cuff was slid up to the base of the tail. For some of the experiments the tails were clamped during the radiation. The internal temperature of the immersed portion of the tail, between the tail vertebrae, was monitored with a thermocouple inserted into a 25-gauge hypodermic needle. The temperature of the immersed portion was found to rise rapidly and become stable at 0·1°C below the water-bath temperature. Mice which had been used for temperature measurements were not included in the analysis.

Skin reactions were assessed 3 times a week for 7 weeks by the skin scoring system shown in Table I. The response was expressed as the average reaction between 15 and 50 days after treatment.

TABLE I.—*Skin scoring system for mouse tails*

| Score | Reaction |
|---|---|
| 0 | No visible reaction |
| 1 | Loss of hair |
| 2 | Slight erythema |
| 3 | Definite erythema |
| 4 | Severe erythema and tail swollen |
| 5 | Severe erythema and dry desquamation |
| 6 | First signs of breakdown |
| 7 | Small areas of moist desquamation |
| 8 | Large patch of breakdown covering 4 mm |
| 9 | Several large patches of breakdown |
| 10 | ¼ of the treated portion with moist desquamation |
| 11 | ½ of the treated portion with moist desquamation |
| 12 | ¾ of the treated portion with moist desquamation |
| 13 | All treated portion with moist desquamation |
| 14 | All treated portion black and withered |
| 15 | All treated portion missing |

### RESULTS

Irradiation alone, up to 15 Gy, caused no visible skin reactions. Doses of 20 Gy or more caused dry desquamation by 20 days after irradiation. Moist desquamation occurred after doses of 25 Gy or more, reaching a maximum at 30 days after exposure, and healing at a rate that was dose-dependent. Typical skin-reaction curves after radiation are shown by the solid lines in Fig. 1. The broken line represents treatment with heat and radiation combined, and is seen to be qualitatively similar to that from radiation alone.

Dose–effect curves for various treatments have been plotted in Fig. 2. It can be seen that, for all the temperatures used, 1 h of hyperthermia potentiates the effects of X-rays. This potentiation may be quantified using the thermal enhancement ratio (TER) (Robinson *et al.*, 1974), which is defined as the dose of X-rays alone divided by the dose in combination with heating which produces the same level of damage. Using a skin reaction of 8 as the reference level of injury, TER values can be calculated from the data in Fig. 2 and are given in Table II for heating under either normal or clamped conditions.

FIG. 1.—The average skin reaction during 50 days after various treatments.

FIG. 2.—The average skin reaction plotted as a function of dose of X-rays. The solid lines represent X-rays before heat. The dashed lines represent heat before X-rays.

× = X-rays alone, ● = 41°C, ▲ = 42°C, ○ = 43°C, ▽ = 43·5°C. Heating was always for 1 h. (a) The mouse tails were not clamped. (b) The mouse tails were clamped only during the irradiation.

TABLE II.—*Thermal enhancement ratios*

| Temperature (°C) | Heat before X-rays | Heat after X-rays |
|---|---|---|
| 42 | 1·91 | 1·35 |
| 43 | 2·98 | 1·91 |
| 43·5 | 3·78 | 2·46 |

We have derived values for the OER (defined as the ratio of the doses to clamped or unclamped tissue to give the same biological effect) from data shown in Fig. 2. Again the skin reaction of 8 was used as the reference level. Table III shows the OER values for tails subjected to hyperthermia and X-rays in either normal or clamped conditions compared with the OER value of 1·77 without hyperthermia.

Although there are no formal methods of estimating the errors on such measurements, an estimate has been obtained by drawing dose–response curves at the outer limits of the experimental data points. From this it seems that the uncertainty of both the OER and TER values is in the region of 5 to 10%.

TABLE III.—*Oxygen enhancement ratios*

| | Heat before clamped X-rays | Clamped X-ray before heat |
|---|---|---|
| Temperature (°C) | Heat before X-rays | X-rays before heat |
| 42 | 2·27 | 1·83 |
| 43 | 2·39 | 1·94 |
| 43·5 | 2·27 | 1·78 |
| Average | 2·31 | 1·85 |

OER without hyperthermia was 1.77.

Normally, mice treated by heat alone had no visible skin reactions or necrosis at any of the temperatures used, except for a slight transient erythema between Days 1 and 3 after the more severe treatments. This occurred whether or not the blood supply was clamped. When the most severe treatment of 43·5°C (which by itself caused no visible damage) was combined with small doses of radiation, there was, however, an occasional incidence of early necrosis, suggesting that radiation enhanced heat damage. Animals showing such early necrosis were excluded from the analysis.

### DISCUSSION

### *Oxygen enhancement ratio*

There are differing reports in the literature on the effect of heat on OER. Using cells *in vitro* Robinson *et al.* (1974) and Kim *et al.* (1975*b*) noted that OER was reduced when hyperthermia was combined with radiation. However, these experiments involved incubating cell suspensions at high density for prolonged periods, a technique which produces an unknown degree of hypoxia and which may cause other changes influencing the hyperthermic response (Durand, 1978). Furthermore, these conditions may produce accumulation of the waste products of metabolism, nutrient deficiency and increased acidity; changes which have been shown to sensitize cells to direct heat damage. Power & Harris (1977) induced hypoxia in cells by passive gas exchange, a technique which reproducibly achieves extreme hypoxia and avoids metabolic stress and medium depletion. In their experiments, hyperthermia at 43°C caused no decrease in OER. Kiefer *et al.* (1976), using cell survival of diploid yeast cells with X-rays and 47°C, also observed no decrease in the OER.

With normal tissues *in vivo*, the effect of heat in enhancing X-ray damage may be separated from direct heat injury. This is very important, because hypoxia may act differently from the enhancement of radiation damage in modifying the direct heat injury. The OER for skin measured without hyperthermia was found to be 1·77. This is lower than the normal values of OER, which lie in the range 2·5–3·0, indicating that, under the experimental conditions used here, the normal skin was slightly hypoxic. Hendry (1978) measured an OER of 1·5 for necrosis of mouse tails at room temperature, the animals being unanaesthetized at the time of the experiment. However, anaesthesia has been shown to increase the radiosensitivity of the mouse tail due to the increased blood flow and consequent increased oxygenation (Hornsey *et al.*, 1977).

When hyperthermia was given after X-irradiation, the average OER for the 3 temperatures used was 1·85 (Table III), very similar to that without hyperthermia. In contrast, when heat was given before X-irradiation the OER increased to an average of 2·3. This increase is probably due to a transient increase in blood circulation following hyperthermia, improving the tissue oxygenation.

However, the possibility of both a decrease in intrinsic OER with improved oxygenation by hyperthermia cannot be totally excluded.

Hendry (1978) also demonstrated improved oxygenation in the mouse tail by warming the animals from room temperature (23–25°C) to 37°C during the irradiation. This increased the OER from 1·5 to 2·0. Using data from Thrall *et al.* (1975) values for the OER have been derived for their endpoint of 50% incidence of dry desquamation on the skin of the mouse leg. In agreement with our results, OER appeared not to change for heat given after irradiation but was significantly greater for heat given first. Also, Myers & Field (1979), using stunting of growth of the baby rat tail, demonstrated a slight increase in OER from 2·0 to 2·3 by prior hyperthermia.

It is therefore our opinion that the combination of hyperthermia and X-rays cannot be considered as a substitute for high LET radiation.

*Thermal enhancement ratio*

It has been reported that the TER for skin damage is greater if heat is given before irradiation (Stewart & Denekamp, 1977). A similar result is seen in Table II where the TER for heat before irradiation is consistently greater than for heat given afterwards. However, the TER values in Table II are probably influenced by the improved oxygenation during irradiation, when heat is given first. An allowance can be made using the measured OER. The TER values thus obtained are still higher than, but more comparable to, those obtained when heat is given after irradiation (Table IV). Our results are shown in Fig. 3, where it can be seen that they are not significantly different from those for other normal tissues, illustrating that in general TER is greater when heat is given first.

TABLE IV.—*Thermal enhancement ratios*

| | Heat first | | Heat after |
|---|---|---|---|
| Temperature (°C) | Measured TER | TER corrected for OER of 2·3 | TER |
| 42 | 1·91 | 1·48 | 1·35 |
| 43 | 2·98 | 2·32 | 1·91 |
| 43·5 | 3·78 | 2·95 | 2·46 |

FIG. 3.—Thermal enhancement ratio for various normal tissues as a function of temperature. The solid lines summarize the data for X-rays before heating (closed symbols) and X-rays after heating (open symbols). Data from Field (1979).
○ ● Skin (present results)
★ Skin (present results corrected for OER of 2·3)
▽ ▼ Skin (Law *et al.*, 1977)
□ ■ Skin (Stewart & Denekamp, 1977)
△ Cartilage (Myers & Field, 1977)
⊟ ■ Intestinal crypts (Hume & Field, 1978)

### CONCLUSIONS

From these experiments, and from analysis of results published elsewhere, it appears that heat does not reduce the OER for X-rays. However, when heat is given before irradiation to a partially hypoxic tissue, there appears to be an increase in OER, and hence also in TER, due to improved oxygenation.

### REFERENCES

DEWEY, W. C., HOPWOOD, L. E., SAPARETO, S. A. & GERWECK, L. E. (1977) Cellular responses to combinations of hyperthermia and radiation. *Radiology*, **123**, 463.

DURAND, R. E. (1978) Effects of hyperthermia on the cycling, noncycling, and hypoxic cells of irradiated and unirradiated multicell spheroids. *Radiat. Res.*, **75**, 373.

FIELD, S. B. (1979) Hyperthermia: Alone or in combination with X-rays? In *Proc. Int. meeting Radio-oncology*, Baden, 1978. Stuttgart: George Thieme (In press).

HAHN, G. M. (1974) Metabolic aspects of the role of hyperthermia in mammalian cell inactivation and their possible relevance to cancer treatment. *Cancer Res.*, **34**, 3117.

HENDRY, J. H. (1978) Radionecrosis of normal tissue: Studies on mouse tails. *Int. J. Radiat. Biol.*, **33**, 47.

HILL, S. A. & DENEKAMP, J. (1978) The effect of vascular occlusion on the thermal sensitization of a mouse tumour. *Br. J. Radiol.*, **51**, 997.

HORNSEY, S., MYERS, R. & ANDREOZZI, U. (1977) Differences in the effects of anaesthesia on hypoxia in normal tissues. *Int. J. Radiat. Biol.*, **32**, 609.

HUME, S. P. & FIELD, S. B. (1978) Hyperthermic sensitization of mouse intestine to damage by X-rays: The effect of sequence and temporal separation of the two treatments. *Br. J. Radiol.*, **51**, 302.

KIEFER, J., KRAFT-WEYRATHER, W. & HLAWICA, M. M. (1976). Cellular radiation effects and hyperthermia. Influence of exposure temperature on survival of diploid yeast irradiated under oxygenated and hypoxic conditions. *Int. J. Radiat. Biol.*, **30**, 293.

KIM, S. H., KIM, J. H. & HAHN, E. W. (1975*a*) Enhanced killing of hypoxic tumour cells by hyperthermia. *Br. J. Radiol.*, **48**, 872.

KIM, S. H., KIM, J. H. & HAHN, E. W. (1975*b*) The radiosensitization of hypoxic tumour cells by hyperthermia. *Radiology*, **114**, 727.

LAW, M. P., AHIER, R. G. & FIELD, S. B. (1977) The response of mouse skin to combined hyperthermia and X rays. *Int. J. Radiat. Biol.*, **32**, 153.

LAW, M. P., AHIER, R. G. & FIELD, S. B. (1978) The response of the mouse ear to heat applied alone or combined with X rays. *Br. J. Radiol.*, **51**, 132.

MORRIS, C. C., MYERS, R. & FIELD, S. B. (1977) The response of the rat tail to hyperthermia. *Br. J. Radiol.*, **50**, 576.

MYERS, R. & FIELD, S. B. (1977) The response of the rat tail to combined heat and X rays. *Br. J. Radiol.*, **50**, 581.

MYERS, R. & FIELD, S. B. (1979) Hyperthermia and the oxygen enhancement ratio for damage to baby rat cartilage. *Br. J. Radiol.*, **52**, 415.

OVERGAARD, J. & BICHEL, P. (1977) The influence of hypoxia and acidity on the hyperthermic response of malignant cells *in vitro*. *Radiology*, **123**, 511.

POWER, J. A. & HARRIS, J. W. (1977) Response of extremely hypoxic cells to hyperthermia: Survival and oxygen enhancement ratios. *Radiology*, **123**, 767.

ROBINSON, J. E., WIZENBERG, M. J. & MCCREADY, W. A. (1974) Combined hyperthermia and radiation suggest an alternative to heavy particle therapy for reduced oxygen enhancement ratios. *Nature*, **251**, 521.

STEWART, F. A. & DENEKAMP, J. (1977) Sensitization of mouse skin to X-irradiation by moderate heating. *Radiology*, **123**, 195.

SUIT, H. D. (1977) Hyperthermic effects on animal tissues. *Radiology*, **123**, 483.

THRALL, D. E., GILLETTE, E. L. & DEWEY, W. C. (1975) Effect of heat and ionizing radiation on normal and neoplastic tissues of the C3H mouse. *Radiat. Res.*, **63**, 363.